# Change in Skin Surface Temperature at the Posterior Neck When Using Acupuncture at Houxi Acupoint in Healthy Volunteers

**DOI:** 10.7759/cureus.52068

**Published:** 2024-01-10

**Authors:** Dieu-Thuong Thi Trinh, Chi-Thien Vo, Minh-Man Pham Bui, Nguyen Lam Vuong

**Affiliations:** 1 Traditional Medicine Administration, Ministry of Health, Hanoi, VNM; 2 Faculty of Traditional Medicine, University of Medicine and Pharmacy at Ho Chi Minh City, Ho Chi Minh, VNM; 3 Department of Medical Statistics and Informatics, Faculty of Public Health, University of Medicine and Pharmacy at Ho Chi Minh City, Ho Chi Minh City, VNM

**Keywords:** posterior neck area, thermographic, skin surface temperature, acupuncture, houxi (si-3) acupoint

## Abstract

Introduction

The use of acupuncture has been suggested for the treatment of neck pain. Recently, a large body of evidence demonstrated that acupuncture has an effect on microcirculation in pain regions, but the exact mechanism remains unclear. This study aims to evaluate the skin surface thermographic changes in the posterior neck associated with manual acupuncture at the Houxi (SI-3) acupoint.

Methods

Sixty healthy volunteers of both genders, aged 18 to 30 years, were randomly determined into two groups: left acupuncture (Group A) and right acupuncture (Group B). Each group underwent two sessions with a seven-day interval. The first session involved acupuncture at the control Yuji (LU-10) acupoint, while the second session featured acupuncture at the SI-3 acupoint. Skin temperature at the posterior neck was measured by using an infrared thermal camera (FLIR C5™, FLIR® Systems, Inc., Wilsonville, OR, USA) at five time points with 5-minute intervals.

Results

There were statistically significant increases in posterior neck skin surface temperature (p < 0.05) during acupuncture at both the left and right SI-3 acupoints, but no significant change was observed during acupuncture at the left and right LU-10 acupoints. Furthermore, acupuncture at the SI-3 acupoint on either hand increased posterior neck skin surface temperature without a statistically significant difference (p > 0.05).

Conclusion

We observed that applying acupuncture at the SI-3 acupoint increased the skin surface temperature of the posterior neck area. Furthermore, the SI-3 acupoint exhibits a uniform impact on the posterior neck area's skin surface temperature, regardless of the side chosen for acupuncture.

## Introduction

Acupuncture, a therapeutic technique in traditional medicine with a history of development spanning over 2500 years, has received acknowledgement from the World Health Organization for its efficacy in treating various pain conditions, including neck pain [1,2]. Acupuncture is also considered to be safer and more effective for neck pain management [3]. Despite its recognition, the precise mechanism of acupuncture remains to be fully elucidated, and how acupoints influence remote areas necessitates rigorous scientific investigation.

The Houxi (SI-3) acupoint is the Shu-stream point of the small-intestine channel, a special point of the 12 meridians that is located distal to the elbows and knees, namely well, brook, stream, river, and sea points. According to its special features, the SI-3 acupoint is an important distal point for pain, stiffness, and contractures along the course of the channel and for disorders of the cervical spine [4]. There are two potential explanations for how SI-3 influences neck pain based on classical traditional medicine texts. The SI-3, as a Shu-stream point, is primarily used to address physical fatigue, joint discomfort, and pain along the SI channel. Furthermore, the SI-3 acupoint serves as the meeting point of the governor vessel, one of the eight extraordinary channels, and is also connected to the bladder channel. Consequently, according to traditional medicine theory, stimulating the SI-3 acupoint can alleviate pain in areas related to the bladder channel and pain along the governing channel, such as headaches, back, leg, and heel pain. Additionally, SI-3 has the capacity to open the governing channel, promote a tranquil spirit (Shen), and clear the mind, all of which can contribute to pain relief [4]. Yet, the specific mechanisms of the SI-3 acupoint remain debated.

Research indicates that microcirculation plays a crucial role in neck pain, with blood flow differing between painful and painless regions. Furthermore, microcirculation, specifically blood flow in neck pain, plays a pivotal role, given the significantly reduced circulation in painful regions compared to pain-free areas [5]. Acupuncture can influence facial blood perfusion (FBP) in remote places connected by meridians [6]. In recent times, advancements in scientific tools have enabled the use of infrared radiation thermometry (IRT) to explore acupoints, making it a non-invasive, cost-effective, and secure method [7,8]. Previous IRT research has primarily concentrated on differentiating acupoints from non-acupoint areas and monitoring temperature shifts at acupoints. Although skin temperature correlates with local microcirculation [9], limited attention has been dedicated to evaluating temperature fluctuations at specific acupoints, a group of acupoints frequently utilized in clinical settings. This study aims to elucidate the relationship between the SI-3 acupoint and the posterior neck area by monitoring changes in skin surface temperature using an infrared camera during acupuncture on healthy volunteers.

## Materials and methods

Study design and participants

This was a trial conducted in the Acupuncture Experimental Research Lab, Faculty of Traditional Medicine, University of Medicine and Pharmacy at Ho Chi Minh City, Vietnam, from October 2022 to April 2023. This study was conducted per the guidelines of the Declaration of Helsinki and approved by the Institutional Review Board of the University of Medicine and Pharmacy at Ho Chi Minh City (approval no. 679/HDDD-DHYD). The protocol has been registered with the Clinical Trial Registry (NCT05581329).

To achieve a power of 0.8 at α value = 0.05, with a 0.574 effect size and potential 10% loss of data, the required size for each group was estimated to be 30 participants [10,11]. Participants criteria included healthy males and females aged from 18 to 30 years old; a body mass index (BMI) of 18.5 to under 23 kg/m2; no problems of stress, anxiety, or depression (according to the depression, anxiety, and stress scale − 21 Items (DASS 21) scale); vital signs within normal limits (pulse 60 to 100 beats per minute, blood pressure from 90/60 to less than 140/90 mmHg, body temperature between 36.16℃ and 37.02℃) [12]; and those not currently participating in other intervention studies. We excluded the following participants: those who had insomnia, common cold, thyroid diseases, inflammation of the neck skin, menstruation period, were pregnant or breastfeeding, performed physical activity two hours before the study, consumed alcohol, coffee, tobacco, sedatives, or drugs that cause vasodilation and affect blood pressure and heart rate within 24 hours before enrolment, used chemical or pharmaceutical products on the posterior neck, and received treatment with physical therapy, heat therapy, cupping, massage, or acupuncture in the posterior neck 24 hours prior to the study.

Study procedure

Recruitment and explanation of procedures were carried out, following which volunteers signed informed consent forms. Eligible subjects were assigned into two groups of 30 (Group A with acupuncture on the left hand and Group B with acupuncture on the right hand) in a 1:1 allocation ratio in a random order. Participants in each group received two sessions seven days apart with the same acupuncture protocol but differing only in acupoint location. In each group, participants received acupuncture at LU-10 in the first session and at SI-3 in the second session. The participants had no knowledge of the location and effect of acupoints and were not informed of their exact acupuncture session sequences.

Acupuncture techniques

**Figure 1:**
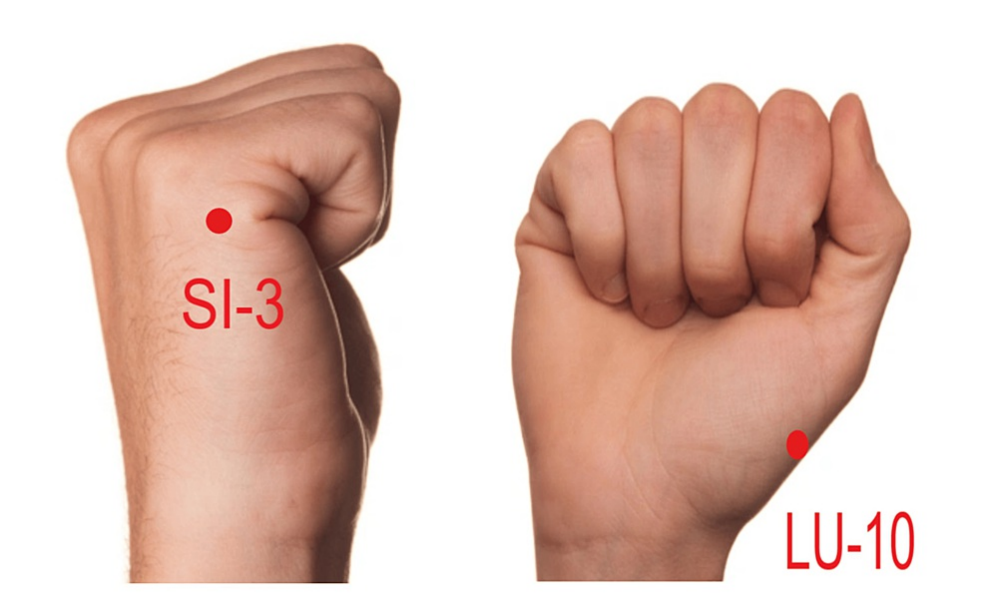
Location of Houxi (SI-3) and Yuji (LU-10) acupoint

In this study, the SI-3 acupoint is determined on the dorsum of the hand, proximal to the ulnar side of the fifth metacarpophalangeal joint, at the demarcation between the red and white flesh [2]. The LU-10 acupoint is located on the palm, radially to the midpoint of the first metacarpal bone, and also at the boundary between the red and white flesh [2] (Figure 1). Acupuncture was performed by a licensed traditional medical doctor with 10 years of experience in acupuncture. Sterile disposable needles (13 mm in length and 0.30 mm in diameter, Khanh-Phong brand) were operated to a depth of 0.5-1 cun on acupoints, depending on the muscle thickness [9].

During every session, the participant sat comfortably on a chair with an exposed hand and skin surface at the posterior neck. Acupuncture was performed by inserting and manipulating the needle for 60 seconds. Upon needle insertion, the practitioner rotated the handle of the needle clockwise and counterclockwise, evenly from 180° to 270°, repeating this action 60 to 120 times within 60 seconds to achieve the Deqi sensation. The needle remained in place for 10 minutes, with additional stimulation every five minutes, and was then removed. The stimulation method was identical for both acupoints and sessions.

Measurements for each acupuncture session

**Figure 2:**
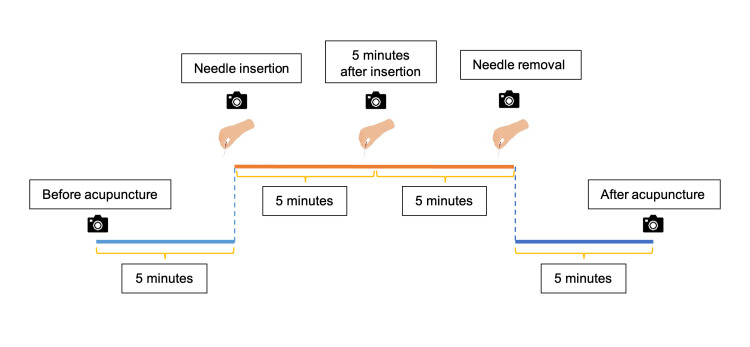
Time points to measure skin surface temperature of the posterior neck

Skin surface temperature at the posterior neck was measured using an infrared thermal camera (FLIR C5™, FLIR® Systems, Inc., Wilsonville, OR, USA) at five time points with 5-minute intervals, i.e., 5 minutes prior to acupuncture (T1), immediately after needle insertion (T2), 5 minutes post-insertion (T3), immediately before needle removal (T4), and 5 minutes after needle withdrawal (T5) (Figure 2). The temperature survey area was the posterior region of the neck (between the inferior borderline of the head and the line across the spinous process of the seventh cervical vertebra and the acromion). The camera was positioned 0.5 meters from the target area, oriented perpendicularly to the skin surface. To account for the effects of circadian rhythms, measurements were taken from 09:00 to 17:00. The room was kept quiet, with the ambient temperature at approximately 25 ± 1°C and the relative humidity at approximately 50% to 60%, without the presence of electrical equipment that generated heat and with no direct incidence of air or sunlight [13,14]. Participants wore comfortable clothes and sat on high-backed chairs with their feet elevated. The FLIR Thermal Studio software, version 1.9 (FLIR® Systems, Inc., Wilsonville, OR, USA), was used to determine the temperature value. Other baseline measurements were taken including pulse, blood pressure, and body temperature by monitoring sheet. Adverse events, such as severe pain, bleeding, nausea, vomiting, and dizziness, were monitored throughout the study.

Statistical analysis

All statistical analyses were performed using the Stata version 14.0 software (STATA Corp., College Station, TX, USA). A p-value of < 0.05 was considered significant. All tests were two-tailed. The continuous variables were expressed as the mean and standard deviation (SD), or median and interquartile range (IQR), whereas the categoric variables were expressed as numbers and percentages. Since the data measured in the present study are not normally distributed, they were used to compare treatment effects on groups at different times, and the Wilcoxon signed-rank test and Mann-Whitney test were used to evaluate differences between the groups.

## Results

General characteristics of the study population

All 60 eligible volunteers, split into 30 per group (A and B), completed the study without any dropouts or exclusions. Table 1 shows the characteristics of the study population. There were no significant differences in gender, age, or BMI between Group A and Group B.

**Table 1:** Characteristics of the study sample at baseline (n = 60) ^*^Tested using chi-square test, ^#^Tested using independent samples T-test SD: Standard deviation, BMI: Body mass index

Characteristics		Group A: Left hand (n = 30)	Group B: Right hand (n = 30)	p-value
Gender, n (%)	Male	15 (50)	15 (50)	1*
	Female	15 (50)	15 (50)	
Age, years (Mean ± SD)		20.97 ± 2.03	20.3 ± 1.91	0.19^#^
BMI, kg/m^2 ^(Mean ± SD)		20.71 ± 1.59	20.42 ± 1.45	0.46^#^

The differences in pulse, blood pressure, and body temperature before acupuncture between the first and second trial sessions were not statistically significant. Besides, there were no statistically significant differences in pulse, blood pressure, and body temperature before and after acupuncture in both groups A and B (Tables 2-3).

**Table 2:** Characteristics of pulse, blood pressure, and body temperature in Group A (n=30) ^*^Comparison between before and after acupuncture; paired samples T-test ^#^Comparison between two trial sessions; independent samples T-test SD: Standard deviation, BPM: Beats per minute

Characteristics		Acupuncture at left Yuji LU-10 acupoint (n = 30)	Acupuncture at left Houxi SI-3 acupoint (n = 30)	p-value
Pulse BPM (Mean ± SD)	Before acupuncture	77.23 ± 8.24	78.33 ± 7.11	0.58^#^
	After acupuncture	76.53 ± 8.21	78.07 ± 7.08	0.44^#^
	p-value	0.17^*^	0.62^*^	-
Systolic blood pressure mmHg (Mean ± SD)	Before acupuncture	106.27 ± 9.59	106.33 ± 9.35	0.98^#^
	After acupuncture	105.33 ± 9.71	105.57 ± 9.62	0.93^#^
	p-value	0.12^*^	0.19^*^	-
Diastolic blood pressure mmHg (Mean ± SD)	Before acupuncture	68.8 ± 6.78	70.17 ± 6.16	0.42^#^
	After acupuncture	69.37 ± 6.63	70.47 ± 6.34	0.51^#^
	p-value	0.27^*^	0.65^*^	-
Body temperature ℃ (Mean ± SD)	Before acupuncture	36.41 ± 0.16	36.47 ± 0.25	0.3^#^
	After acupuncture	36.44 ± 0.17	36.48 ± 0.2	0.44^#^
	p-value	0.23^*^	0.86^*^	-

**Table 3:** Characteristics of pulse, blood pressure, and body temperature in Group B (n=30) ^*^Comparison between before and after acupuncture; paired samples T-test ^#^Comparison between two trial sessions; independent samples T-test SD: Standard deviation, BPM: Beats per minute

Characteristics		Acupuncture at right Yuji LU-10 acupoint (n = 30)	Acupuncture at right SI-3 acupoint (n = 30)	p-value
Pulse BPM (Mean ± SD)	Before acupuncture	75.9 ± 9.07	77.47 ± 7.54	0.47^#^
	After acupuncture	75.87 ± 9.12	77.5 ± 7.94	0.46^#^
	p-value	0.95^*^	0.96^*^	-
Systolic blood pressure mmHg (Mean ± SD)	Before acupuncture	108.73 ± 10.5	106.87 ± 8.67	0.46^#^
	After acupuncture	107.87 ± 9.44	107.33 ± 7.07	0.81^#^
	p-value	0.29^*^	0.55^*^	-
Diastolic blood pressure mmHg (Mean ± SD)	Before acupuncture	70.83 ± 6.74	71.17 ± 7.49	0.87^#^
	After acupuncture	70.77 ± 7.39	72.33 ± 6.42	0.38^#^
	p-value	0.97^*^	0.11^*^	-
Body temperature ℃ (Mean ± SD)	Before acupuncture	36.43 ± 0.13	36.46 ± 0.17	0.41^#^
	After acupuncture	36.47 ± 0.16	36.49 ± 0.2	0.51^#^
	p-value	0.19^*^	0.36^*^	-

Temperature difference between time points

**Figure 3:**
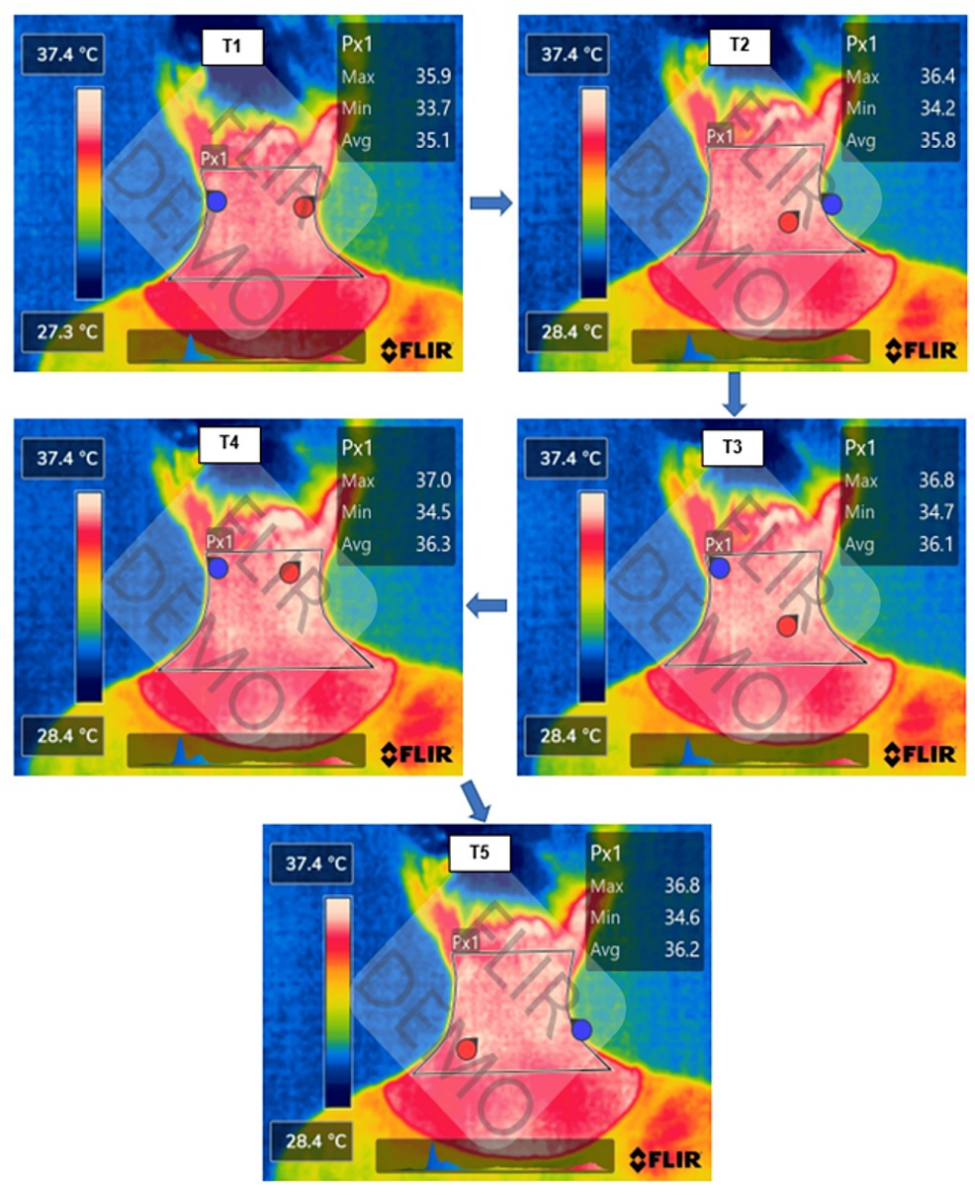
The change in skin surface temperature when acupuncture was done at the Houxi acupoint

When acupuncture was done at the left and right LU-10 acupoints, there was no difference in skin surface temperature in the posterior neck between the times of T2, T3, T4, and T5 compared to T1. However, we observed statistically significant differences in skin surface temperature of the posterior neck at the time of T2, T3, T4, and T5 compared to T1 (p < 0.01) when acupuncture was done at the left and right SI-3 acupoints (Table 4 and Figure 3).

**Table 4:** The temperature difference between the time points after acupuncture compared to T1 *Comparison between before and after acupuncture; paired samples T-test T1: 5 minutes before acupuncture, T2: Immediately after insertion, T3: After 5 minutes of insertion, T4: Immediately before needle withdrawal, T5: 5 minutes after needle withdrawal

Time-point comparisons	Group A		Group B	
	p-value (LU-10)	p-value (SI-3)	p-value (LU-10)	p-value (SI-3)
T2 compares with T1	0.59^*^	< 0.01^*^	0.34^*^	< 0.01^*^
T3 compares with T1	0.68^*^	< 0.01^*^	0.5^*^	< 0.01^*^
T4 compares with T1	0.76^*^	< 0.01^*^	0.42^*^	< 0.01^*^
T5 compares with T1	0.69^*^	< 0.01^*^	0.38^*^	< 0.01^*^

Temperature differences between acupuncture at LU-10 and SI-3

**Figure 4:**
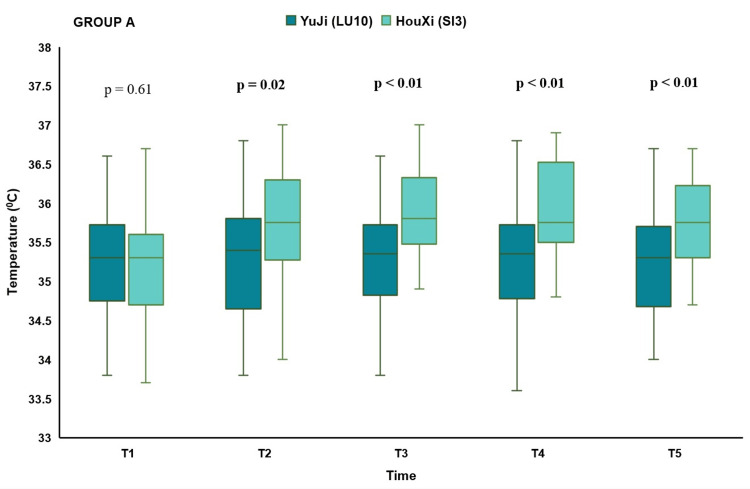
Differences in skin surface temperature of the posterior neck between acupuncture at LU-10 and SI-3 on the left side in Group A

In Group A, at baseline, there was no difference in the skin surface temperature of the posterior neck before acupuncture between the two sessions. At the time after acupuncture at T2, T3, T4, and T5, the median skin surface temperature of the posterior neck was 35.4℃ IQR (34.7−35.8), 35.35℃ IQR (34.9−35.7), 35.35℃ IQR (34.8−35.7), 35.3℃ IQR (34.7−35.7), resepectively, at the left LU-10 acupoint, and 35.75℃ IQR (35.3−36.3), 35.8℃ IQR (35.5−36.3), 35.75℃ IQR (35.5−36.5), 35.75℃ IQR (35.3−36.2), resepectively, in participants with acupuncture at the left SI-3 acupoint. The difference in skin surface temperature of the neck at the post-insertion times between acupuncture at LU-10 and SI-3 was statistically significant. Compared with acupuncture at the left LU-10, participants with acupuncture at the left SI-3 acupoint had a statistically significant higher skin surface temperature of the posterior neck at the post-insertion times of T2, T3, T4, and T5 (p < 0.01) (Figure 4).

**Figure 5:**
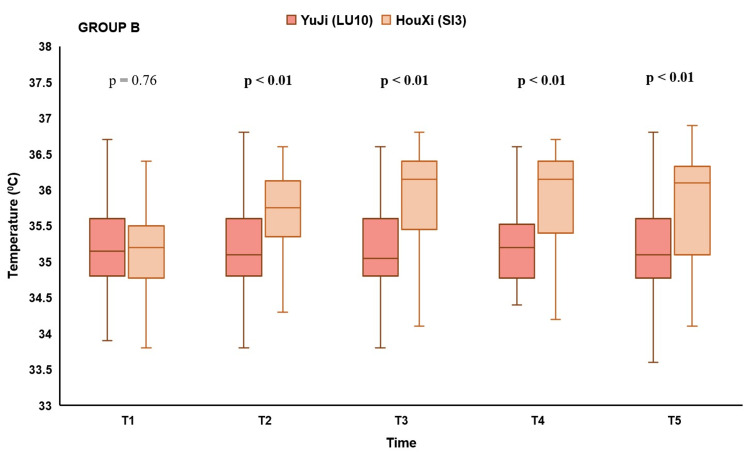
Differences in skin surface temperature of the posterior neck between acupuncture at LU-10 and SI-3 on the right side in Group B

In Group B, at baseline, there was no difference in the skin surface temperature of the posterior neck before acupuncture between the two sessions. At the time after acupuncture at T2, T3, T4, and T5, the median skin surface temperature of the posterior neck was 35.1℃ IQR (34.8-35.6), 35.5℃ IQR (34.8 - 35.6), 35.2℃ IQR (34.8 - 35.5), 35.1℃ IQR (34.8 - 35.6), respectively, at the right LU-10 acupoint, and 35.75℃ IQR (35.4 - 36.1), 36.15℃ IQR (35.5 - 36.4), 36.15℃ IQR (35.4 - 36.4), 36.1℃ IQR (35.1 - 36.3), respectively, in participants with acupuncture at the right SI-3 acupoint. The difference in skin surface temperature of the neck at the post-insertion times between acupuncture at LU-10 and SI-3 was statistically significant. Compared with acupuncture at the right LU-10, participants with acupuncture at the right SI-3 acupoint had a statistically significant higher skin surface temperature of the posterior neck at the post-insertion times of T2, T3, T4, and T5 (p < 0.01) (Figure 5).

When acupuncture was done at the SI-3 acupoint on the left hand and the right hand, the difference in skin surface temperature of the posterior neck was not statistically significant between the two groups (Table 5). 

**Table 5:** Comparison of posterior neck temperature when acupuncture was done at the left and right Houxi (SI-3) acupoints ^a^ Mann-Whitney test, T1: 5 minutes before acupuncture, T2: Immediately after insertion, T3: After 5 minutes of insertion, T4: Immediately before needle withdrawal, T5: 5 minutes after needle withdrawal IQR: Interquartile range

Times	Group A: Left hand (n = 30)	Group B: Right hand (n = 30)	p-value
Temperature at T1, ℃ Median (IQR)	35.3 (34.7−35.6)	35.2 (34.8−35.5)	0.69^a^
Temperature at T2, ℃ Median (IQR)	35.75 (35.3−36.3)	35.75 (35.4−36.1)	0.75^a^
Temperature at T3, ℃ Median (IQR)	35.8 (35.5−36.3)	36.15 (35.5−36.4)	0.56^a^
Temperature at T4, ℃ Median (IQR)	35.75 (35.5−36.5)	36.15 (35.4−36.4)	0.93^a^
Temperature at T5, ℃ Median (IQR)	35.75 (35.3−36.2)	36.1 (35.1−36.3)	0.68^a^

Adverse events

There were three cases of bleeding after withdrawing the needle. The level of bleeding was not serious and was managed by pressing and holding a sterile cotton swab at the insertion site for 30 seconds.

## Discussion

The study's primary finding revealed a significant increase in skin surface temperature at the posterior neck after acupuncture-induced Deqi at both left and right SI-3 acupoints. However, under similar conditions, when acupuncture and stimulation were applied to the LU-10 acupoint, no change in skin temperature was observed. This outcome provides concrete evidence of the corresponding relationship between the SI-3 acupoint and the posterior neck area, as confirmed by prior research on temperature changes in specific effect areas corresponding to acupoints [10,15,16].

Our hypothesis posits that the rise in skin surface temperature at the posterior neck is linked to enhanced microcirculation beneath the skin in this region. In thermoregulatory physiology, roughly 60% of heat dissipation occurs through infrared radiation in resting conditions, reliant on core body heat transfer via the circulatory system to the skin's periphery, subsequently radiated as heat. This process is directly proportional to subcutaneous blood flow. Studies by Huang et al. [17] and others have shown that acupuncture at certain acupoints can elevate microcirculation at related acupoints. For example, acupuncture at the Quchi acupoint (LI-11) increased blood flow at the Sanjian (LI-3), Hegu (LI-4), and Yangxi (LI-5) acupoints. Similar findings for the 'six major acupoints' group corroborate the specificity of acupoints in modifying blood flow within defined areas, as demonstrated in the studies of Wang et al. [18] and Tian et al. [6]. These collective outcomes further substantiate our hypothesis.

Prior studies have indicated an increase in nitric oxide (NO) synthesis within stimulated meridians and acupoints at the biochemical level. In a study conducted by Tsuchiya et al. [19], it was observed that acupuncture led to enhanced blood flow and increased NO concentration in the treated limb, showing a positive correlation. This suggests that the elevation in NO concentration contributes to heightened blood circulation, subsequently increasing heat dissipation through skin radiation. However, further research is necessary to substantiate this hypothesis.

Moreover, when acupuncture was applied to the SI-3 acupoint on either the right or left hand, there was an equivalent increase in skin surface temperature on both sides of the posterior neck. This observation provides initial insights into the bilateral SI-3 acupoints having a uniform effect on the posterior neck region, aligning with traditional medicine theories about meridians and acupuncture points. In traditional medicine, stream (Shu) points are areas where the Qi of the channel flows and swirls, leading to a rapid and substantial Qi flow, earning them the name 'transporting' points. Furthermore, these points are where defensive Qi congregates. The SI-3, functioning as the Shu point of the small-intestine meridian, experiences flourishing Qi in the meridian. Additionally, it serves as the opening point of the governing vessel (Du Mai). As a result, acupuncture-induced Deqi at the SI-3 acupoint promotes Qi movement, not only in the small-intestinal meridian but also within the governing vessel. The governing vessel, often referred to as the 'sea of Yang channels', influences all Yang channels, reinforcing the body's Yang energy. According to certain traditional medicine theories, skin surface temperature is regulated by defensive Qi, implying that eliciting a Deqi sensation through stimulation can modulate skin temperature accordingly [4].

Our study findings offer evidence supporting the selection of remote acupoints, distant from the affected area, for treatment. Additionally, the uniform effect on posterior neck temperature by both left and right SI-3 acupoints suggests that these acupoints share comparable therapeutic effects on the posterior neck. This flexibility allows for the alternating use of either SI-3 acupoint in clinical treatment, tailored to the specific patient's condition.

Regarding safety, only three cases of bleeding following needle removal were observed in our study, and they were minor with no further complications. This aligns with prior research, reinforcing the notion that acupuncture is a relatively safe medical treatment with infrequent serious side effects. Most complications are mild, such as pain, bleeding, or swelling, requiring no treatment [20].

These complications typically arise from factors like the patient's mental stress, improper acupuncture manipulation, or insufficient disinfection of the acupuncture area [21]. To mitigate these risks, we conducted thorough assessments of participants' medical history, vital signs, and allergy history. Our acupuncturist, holding a medical doctor's license and specialized training in acupuncture, rigorously adhered to the prescribed technical procedures issued by the Ministry of Health, closely monitoring participants during the procedure. These measures contributed to the low incidence of complications during acupuncture.

According to Fernández-Cuevas et al. [13], factors influencing infrared camera temperature survey images include environmental, personal, and technical factors. Our study addressed personal factors by ensuring the homogeneity of age, gender, and BMI between study groups. Both LU-10 and SI-3 acupoints were examined in the same group, eliminating variations related to these personal factors.

Physiological parameters like pulse, blood pressure, and body temperature exhibited no statistically significant differences between the first and second sessions, reinforcing the volunteers' consistent physiological states during the trials. Post-acupuncture measurements revealed no significant alterations in pulse, blood pressure, or body temperature, confirming the safety of acupoint application in clinical settings, particularly for patients with pre-existing cardiovascular conditions. The lack of significant changes in body temperature before and after acupuncture in both study sessions reinforces the specificity of any observed temperature changes in localized areas.

This pioneering study utilized infrared camera technology to investigate the corresponding relationship between the SI-3 acupoint and the posterior neck. Distinguished by its comprehensiveness, this research introduces a non-specific acupoint control group while employing single blinding to enhance result accuracy. Rigorous standards were adhered to for the research environment and participants, with explicit evaluation of adverse events, thereby bolstering evidence of acupuncture safety for clinical applications.

While this study aims to assess the association between the SI-3 acupoint and posterior neck skin surface temperature, the specific mechanisms underlying the temperature increase remain elusive. Further exploration and evaluation of acupoint effects using alternative methodologies is warranted. Additionally, due to the limitations of the infrared camera equipment, continuous monitoring of skin surface temperature fluctuations was not possible. Hence, we recommend conducting similar investigations with equipment capable of continuous temperature recording to gauge the change in skin surface temperature when acupuncture is done at the Houxi acupoint.

## Conclusions

The study's findings provide both objective evidence and therapeutic implications for the effectiveness of the SI-3 acupoint in treating the posterior neck area by examining the association between the SI-3 acupoint and posterior neck skin surface temperature. Furthermore, this research indicates that applying acupuncture to either the left or right SI-3 acupoints exhibited comparable therapeutic effects on posterior neck skin surface temperature. Besides, acupuncture at the Houxi acupoint is safe when applied clinically if specific acupuncture procedures are followed.
